# Risk Pathways for Gonorrhea Acquisition in Sex Workers: Can We Distinguish Confounding From an Exposure Effect Using A Priori Hypotheses?

**DOI:** 10.1093/infdis/jiu484

**Published:** 2014-12-01

**Authors:** Gabriela B. Gomez, Helen Ward, Geoffrey P. Garnett

**Affiliations:** 1 Department of Global Health and Amsterdam Institute for Global Health and Development, AMC, University of Amsterdam, The Netherlands; 2 Department of Infectious Disease Epidemiology, School of Public Health, Imperial College London, United Kingdom; 3 HIV Department, Bill & Melinda Gates Foundation, Seattle, Washington

**Keywords:** framework, gonorrhea, sex workers, risk of acquisition

## Abstract

The population distribution of sexually transmitted infections (STIs) varies broadly across settings. Although there have been many studies aiming to define subgroups at risk of infection that should be a target for prevention interventions by identifying risk factors, questions remain about how these risk factors interact, how their effects jointly influence the risk of acquisition, and their differential importance across populations. Theoretical frameworks describing the interrelationships among risk determinants are useful in directing both the design and analysis of research studies and interventions. In this article, we developed such a framework from a review looking at determinants of risk for STI acquisition, using gonorrhea as an index infection. We also propose an analysis strategy to interpret the associations found to be significant in uniform analyses of observational data. The framework and the hierarchical analysis strategy are of particular relevance in the understanding of risk formation and might prove useful in identifying determinants that are part of the causal pathway and therefore amenable to prevention strategies across populations.

The nature of causation has been the subject of a comprehensive debate in philosophy, medicine, and, relatively more recently, epidemiology. The 2 main interpretations of causation that have been explored are (1) a deterministic interpretation, whereby a cause is invariably followed by its effect, being both necessary and sufficient; and (2) a probabilistic interpretation, where a cause increases or decreases the likelihood of the effect occurring, but is not required to be necessary or sufficient [1, 2]. In this article, we aim to provide a plausibility and probability assessment of the available literature to develop risk pathways, taking as an example sexually transmitted infections (STIs) in general and gonorrhea in particular.

Sexually transmitted infections, including treatable bacterial infections such as gonorrhea, represent a significant global health burden [3]. Eighty percent of women and up to 10% of men remain asymptomatic during gonorrhea infection; when symptomatic, patients present with inflammation of the urogenital tract, throat, or rectum producing discomfort and discharge. Possible complications include pelvic inflammatory disease leading to infertility and ectopic pregnancy. The presence of an untreated STI also enhances human immunodeficiency virus (HIV) acquisition and transmission risks [3–5]. Whereas the deterministic (necessary and sufficient) cause of gonorrhea infection at an individual level (ie, the presence of the bacterium *Neisseria gonorrhoeae*), is well described; at a population level, probabilistic causes that determine the variability in the population distribution of the infection and their complex interplay are less well understood [5].

The spread of STIs, including gonorrhea, in a population can be understood within the basic reproductive number (R_0_) framework, where R_0_ depends on 3 components: (1) β, a measure of infectivity or transmissibility; (2) c, a measure of the contact rate between susceptible and infected individuals; and (3) D, a measure of the duration of infectiousness. The heterogeneous distribution of these determinants and behaviors among individuals, across populations, and over time explains why STIs behave differently within and between populations. The theoretical framework provided by mathematical models allows us to integrate biological and behavioral data to improve our understanding of the distribution of STIs in populations. Each of these 3 determinants are driven by a complex range of factors (social, economic, demographic, cultural, and behavioral) and at a number of levels (in the individual, within a partnership, and in the community). The key questions are which ones are driving an epidemic in a specific context and how we can describe the interconnectedness of different levels and among different factors to explain the causal pathways that lead to an individual being at risk of acquiring or transmitting an STI. In other words, what are the risk factors influencing each component of R_0_ directly, and what are the broader risk factors that must be altered to influence the spread of infection in a population?

The standard method for identifying risk factors in epidemiological research involves comparing exposures in those with and without infection or comparing the incidence of infection in those with or without an exposure in observational studies. These methods have played an important and successful role in understanding many noncommunicable diseases, and have been widely used to explore risk factors for acquiring STIs. However, the intrinsic limitation of risk factor epidemiology [6] is that an enumeration of associations is just the start of the interpretation of a process leading to disease. An explanation requires a hierarchy in the association to develop understanding [2]. Additionally, the selection made by researchers of which risk factors are measured or included in the analyses is often an unclear process and one that may limit the interpretation of the results.

In the case of gonorrhea infection, questions remain about how risk factors interact, how their effects jointly influence the risk of acquisition, and their differential importance across populations. The understanding of the links between these variables will then have both programmatic and policy implications. The decision of where to target an intervention should be guided by this understanding in a particular population and, therefore, in the identification of “drivers” (ie, those determinants with the biggest potential for risk reduction), so that prevention efforts could be directed toward modifiable risk factors within the risk formation pathway in specific vulnerable subgroups—for example, an improvement of healthcare access in defined geographical areas or a reduction of high-risk sexual behaviors within characterized subpopulations.

To date, there have been several frameworks proposed to specify the relations among variables to explain and/or predict an individual's or a group of individuals' risk [1, 2]. In general models of health and illness, such as the socioecological framework [7] or the determinants of health framework [8], the main outcome is a general ill health. In HIV epidemiology research, 2 theoretical frameworks available are the social epidemiology framework [9] and the proximate determinants frameworks [10].

The social epidemiology framework proposes a hierarchy of variables, with transmission dynamics nested within individual characteristics, which are in turn nested within a social context and then in a structural level. This representation of causal pathways illustrates the complexity of mechanisms of causation and how small our understanding is. The proximate determinants framework was originally developed for the study of fertility and child mortality and was adapted by Gregson et al in 1997 and Boerma et al in 2003 to the HIV epidemic in sub-Saharan Africa [11–14]. In 2005, Boerma and Weir summarized it [10], defining pathways of risk, whereby the underlying determinants (sociocultural, economic, and program characteristics) influence the proximate determinants (behavioral and biological components). The proximate determinants, in turn, have direct links to the biological determinants, which affect the rate of new infections. Furthermore, the linear risk structure of the proximate determinants framework can be statistically tested [15].

For a curable STI such as gonorrhea, we can expect the determinants involved in the epidemic to be similar to those shaping HIV epidemiology. However, the relative importance of determinants may differ due to the differences in natural history and influence of healthcare, implying that while different strategies or targets might be needed for prevention and control programs, a similar conceptual framework could be brought to bear in the formulation of a priori hypotheses. In this study, we build on the 2 frameworks using the hierarchical structure of the social epidemiology framework while applying the linear risk structure of the proximate determinants framework to test our hypotheses and structure the evidence available on risk factors for gonorrhea acquisition in female sex worker populations. We present a strategy for interpretation of observational data analysis to assess pathways of risk. We reviewed the literature to identify factors statistically associated with gonorrhea, and analyzed results aiming to distinguish determinants on the risk formation pathway (which are therefore useful to the targeting of intervention) from determinants not on the risk formation pathway that might be considered confounders (for which adjustment is imperative but targeting of an intervention might prove futile). In brief, studies published since 1989 were identified through searches of PubMed/Medline with no restrictions on language. Articles were retrieved by searching “prostitute/prostitution/sex work/sex worker,” with either “gonorrhoea/gonorrhea” or “sexually transmitted infection/STI/sexually transmitted disease/STD.” We included publications of primary research only that assessed urogenital gonorrhea by biological tests only. Studies were included if they calculated an effect estimate for risk factors on gonorrhea or if they provided enough information on their distribution for this to be calculated (more detail is given in the Supplementary Data).

## A THEORETICAL FRAMEWORK OF RISK FOR GONORRHEA ACQUISITION

**Figure 1.**
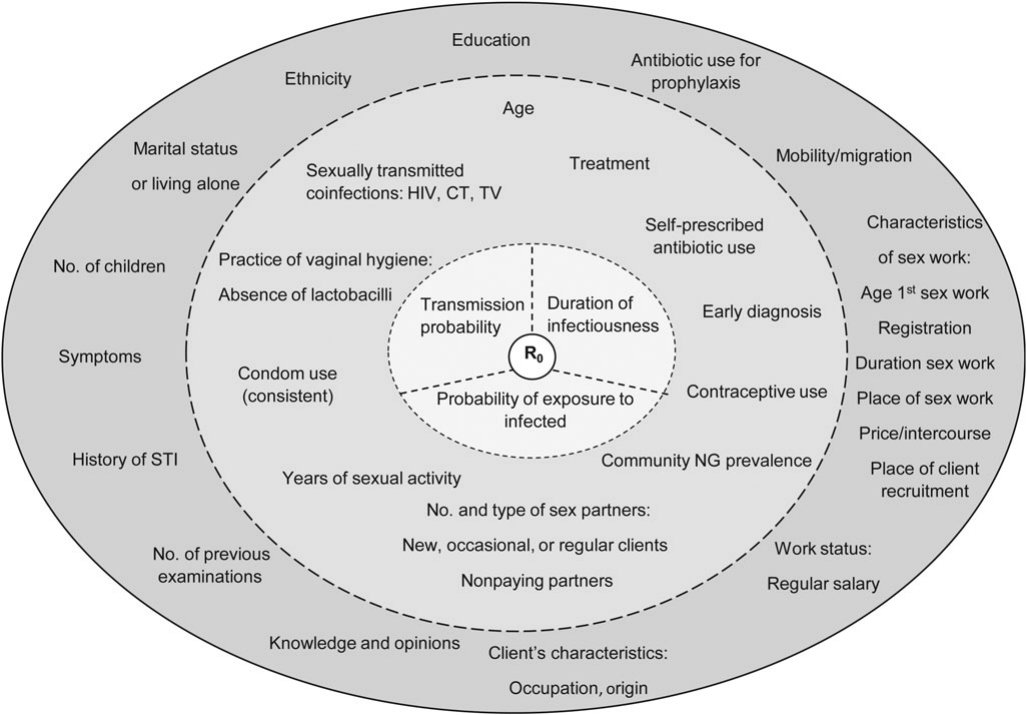
Proposed framework of gonorrhea risk from studies of female sex worker populations. R_0_, the reproductive number, is at the center of the figure. At the first level, directly influencing R_0_, are its 3 components: probability of exposure to infected, transmission probability, and the duration of infectiousness. At a second level, we positioned all the proximate determinants that influence each of the components of R_0_. At a third level, there are the distal/underlying determinants. When different types of measures or proxies of the same determinant were found in the literature, we indicated the determinant and proceeded to list the different measures found (eg, characteristics of sex work: age at first sex work, duration of sex work, registration, place of sex work, place of client recruitment, price per intercourse). Abbreviations: CT, *Chlamydia trachomatis*; HIV, human immunodeficiency virus; NG, *Neisseria gonorrhoeae*; No, number; R_0_, reproductive number; STI, sexually transmitted infection; TV, *Trichomonas vaginalis*.

The findings of our review are summarized in Figure 1. As mentioned above, at a population level, the spread of an infection is defined by its R_0_, representing the average number of new cases generated by 1 primary case [5]. It is implied that the higher the R_0_, the greater the potential for the infection to spread (Figure 1). However, it is worth noting that whereas R_0_ is theoretically related to the potential for spread through the population, the reviewed studies relate to the risk of an infection occurring in the individual. Theoretically, this will be determined by the force of infection that combines the transmission probability, number of contacts, and prevalence of infection in these contacts [βc(ninfected/total population)], along with the duration of infection and the associated chance of an infection remaining at the period of sampling (access to treatment and healthcare). Thus, the importance of the 3 components of R_0_ differs from the reason they are central in our framework for risk, but the correspondence is useful in translating findings from the individual to the population. Subsequently, risk factors found in the literature were organized in 2 levels (underlying and proximate) influencing the 3 components of R_0_: probability of transmission, probability of exposure to an infected person, and average duration of infectiousness [5, 9, 10].

## INTERPRETATION OF OBSERVATIONAL DATA ANALYSES

**Figure 2.**
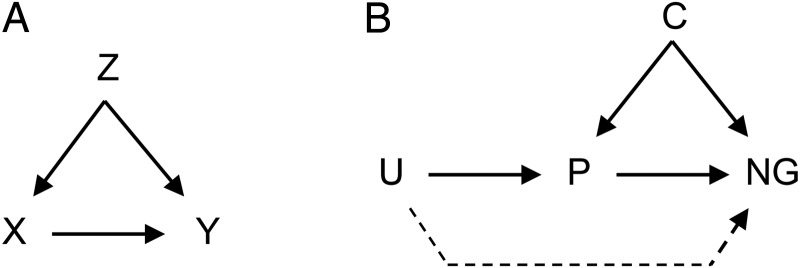
Illustration of confounding. Abbreviations: C, confounder; NG, *Neisseria gonorrhoeae* (outcome); P, proximate risk factor; U, underlying risk factor; X, explanatory variable; Y, outcome; Z, confounder. (See text for details.)

In traditional analyses of observational studies, researchers explore bivariable associations between the outcome and a range of variables explaining the outcome. This analysis is followed by the construction of multivariable statistical models aiming to control for confounding, frequently assuming that all determinants operate at the same level (in terms of proximity to risk, ie, proximate vs underlying). Confounding is therefore defined statistically as a correlation between variables (Figure 2*A*): “X and Y are confounded when there is a third variable, Z, that correlates with both X and Y” [16]. Conclusions of these studies will be sensitive to how many and which explanatory variables (X) and confounders (Z) are included to explain the variability of the outcome (Y), as well as how accurately they are measured.

By expanding this perspective and introducing a priori hypotheses, we move from a rigorous statistical definition aimed at identifying valid and tightly associated determinants, to a broader view using previous knowledge and assumptions to construct plausible pathways linking risk factors. As illustrated in Figure 2*B*, assuming that an “underlying” risk factor (U) is one that influences the outcome (*Neisseria gonorrhoeae*) via a “proximate” risk factor (P), then if the proximate determinants are sufficiently well measured, the effect of underlying determinants should be removed [15, 17] once the model includes proximate determinants. If the proximate determinants are not sufficiently well measured, we will observe a correlation between the underlying determinant and the outcome (in Figure 2*B*, this unmeasured effect is illustrated by a dashed arrow). In this case, we included qualitative assumptions to differentiate between the variable (U) on the causal pathway from a confounder (C).

### Determinants and Associations

In Table 1 we present the interpretation we followed in the classification of determinants as being proximal or underlying when reassessing the results of studies selected. Variables that fall into the proximate determinants category can be classified into those describing biological characteristics, individual behavior, and partner's characteristics. Underlying determinants are those describing sociodemographic characteristics of the individual, characteristics of sex work, and individual knowledge and attitude.

**Table 1.** Interpretation of Different Multivariable Models

In Multivariable Models	Interpretation
Proximate determinants	Effect of proximate determinants adjusted for other proximate determinants
Underlying determinants	Effect of underlying determinants adjusted for other underlying determinants
Proximate and underlying determinants	Effect of proximate determinants adjusted for other proximate determinants
	Effect of underlying determinants adjusted for other underlying determinants and not mediated by proximate determinants

Source: Lewis et al [15], Victora et al [17].

#### Probability of Exposure to Infected Individuals

As with other infections, one individual's outcome is dependent upon the outcomes and exposures of others. Having unprotected sex with an infected partner is an axiomatic prerequisite for infection and thus, the most accurate predictor of acquisition risk defining the value of the probability of exposure to infected in relation to R_0_. Yet partner's infectious status is rarely available. Therefore, researchers measure proxy risk factors to estimate the probability of exposure to an infected person. For instance, the prevalence of the infection in the community constitutes a population-level proximate determinant of the probability of exposure.

Characteristics of sex work and clients, mobility, and prophylactic use of antibiotics were all underlying determinants related to the acquisition risk of gonorrhea and specific to female sex worker populations, affecting the value of probability of exposure to infected in relation to R_0_. In particular, sex work characteristics are an important set of underlying determinants, determining number and type of clients, as well as exposure to poverty and marginalization, which in turn will be related to an increase in violence and less empowerment to negotiate safer sex practices. Self-medication when symptomatic might delay access to healthcare, whereas prophylactic use of antibiotics was included as an underlying determinant as it might give a false sense of security and preclude condom use.

#### Transmission Probability

The transmission probability, directly in relation to R_0_, varies from one pathogen to another. In the case of *Neisseria gonorrhoeae*, it is estimated to be high and modified by individual-level determinants. Transmission from male to female seems to be more efficient than from female to male. Young women are more susceptible to infection than older women, through the same biological pathway observed due to the use of oral contraceptive and injectable hormones, whereby cervical ectopy increases the risk of acquiring gonorrhea and is more common among hormonal contraceptive users and younger women [18].

Coinfections have also been found to increase the transmission probability, whereas barrier methods, such as condoms, are effective in decreasing the risk for gonorrhea transmission or acquisition in particular and STIs in general. In a vulnerable population, such as female sex workers, the risk of STI acquisition is modified by a consistent use of condoms. Condom use in turn varies depending on the type of sexual partner. It has been observed to be less consistent with partners or regular clients than new or occasional clients, implying that only the partners with whom female sex workers do not use condoms are increasing their risk. Therefore, in a population such as female sex workers, the number of partners might be a less accurate measure of risk than the type of partner, which in turn will determine the sexual behavior.

#### Duration of Infectiousness

Early diagnosis and effective treatment are the most efficient approaches to reduce the duration of infectiousness (in direct relation to R_0_) and, as such, were classified as proximate. These are influenced by the presence of biological symptoms, health beliefs, and community and structural determinants of access to healthcare, such as structure of healthcare systems and measures of marginalization or social conflict.

### Framework Application

As an example, we look at condom use and number and characteristics of sexual partners, which are factors associated with transmission probability and probability of exposure, which in turn define R_0_. Klausner et al (Philippines), Sanchez et al (Peru), Pettifor et al (Madagascar), and van den Hoek et al (China) all found that inconsistent condom use with clients was significantly associated with gonorrhea risk in univariable and multivariable analyses [19–22]. Thus, nonconsistent condom use can be considered as a proximate determinant (odds ratio [OR]/risk ratio, 1.5–3). Whether or not condom use is on the causal pathway will be explored by seeing if there is a change on the crude effect of other variables after including condom use in the models. Van den Hoek et al found that the number of clients per week was significant in univariable analysis, and became not significant when added to a model including condom use and current coinfections [21]. Klausner et al reported similar results: Determinants such as the number of regular clients in the past month (>1) and new clients in the last week (≥2) were significant in univariable analysis but then became not significant in the multivariable model including condom use [19]. However, having had sex with a new client in the past month became significant in multivariable analysis after being not significant in univariable analysis.

In this population, the number of sexual partners is a distal determinant in the causal pathway. The risk is derived from the sexual behavior (consistent condom use) with those partners independently of their number, which in turn is predicted by their characteristics.

A second example is self-prescribed prophylaxis and vaginal hygiene, which are associated with duration of infectiousness and transmission probability, directly affecting R_0_. Klausner et al were the only authors looking at the use of antimicrobials or other treatment self-prescribed as prophylaxis [19]. It increased the risk of gonorrhea infection in univariable analysis (OR, 2.1 for prophylactic use during the last month) but became not significant in multivariable analysis including social and structural determinants. However, self-prescription of STI prophylaxis in the past week was significant and increased the risk of gonorrhea in both univariable (OR, 3.2) and multivariable analyses (adjusted OR, 2.5). It might be that, in this population, the effect of antibiotic prophylaxis on gonorrhea risk was measured by behavioral determinants such as condom use, number of clients, or vaginal hygiene practices. Once these variables were added to the model, prophylactic use of antibiotics became not significant. These behavioral determinants may be related to suspicion of a partner's infection and be part of a risk reduction strategy that includes antibiotic prophylaxis. The authors also suggested a mechanism by which regular antibiotic use might have an effect on vaginal flora by suppressing lactobacilli, which might explain why recent (last week) but not early (last month) antimicrobial use remains significant in multivariable analysis. The use of antibiotics for STI symptoms in the past month or week, self-prescribed or not, was not significant.

Routine vaginal hygiene practices, particularly the use of domestic products, were associated with a reduction in the risk of acquiring gonorrhea both in univariable and multivariable analysis in the same study of Klausner et al [19]. Indeed, genital cleansers (shampoos, detergents, and toothpaste) containing an alkyl sulphate surfactant, sodium dodecyl sulphate, might have an inhibitory effect on genital pathogens, decreasing transmission rates [23]. It can therefore be argued that practicing vaginal hygiene is a proximate determinant of gonococcal risk that alters both vaginal pH and flora, being a determinant for per-contact transmission probability, and that its prevalence is influenced by sociocultural determinants.

## DISCUSSION

We categorized risk factors for gonorrhea acquisition reported in 36 studies according to their proximity in the causal pathways to understand risk formation in a population at higher risk. The aim was to explore and explain an analysis strategy moving from a uniform to a hierarchical account of disease causation. In so doing, a critique of the reporting of observational studies of gonorrhea infection is offered. The picture that emerges of the risk of gonorrhea acquisition is that these theoretical frameworks are justified, but only through glimpses they provide at the underlying pathways leading to risk. These pathways should be the target of effective interventions. The use of “proximate/distal” nomenclature has been previously criticized, as it might imply notions of time or distance that are not part of the analysis of observational data [24]. However, it can be useful for researchers to adopt a hierarchical approach to the analysis and interpretation of the data to test hypotheses while recognizing the limitations of the linear image to drawing the whole picture.

This review employed a systematic approach to minimize biases and by going beyond traditional risk factors, the theoretical framework proposed offers a broad picture of the relationships between social, cultural, and individual determinants. However, it also presented limitations. The number of studies included was small compared with the number of publications identified. Additionally, by limiting this review to published articles, we aimed to avoid poor-quality studies, which might have led to some publication bias.

The quality of reporting of the studies included was mixed. Few studies reported a wide range of variables. None of them explored causal pathways explicitly. An ideal analysis of STI risk determinants should include a hierarchical approach based on the framework proposed. This will help avoid distal factors being inappropriately adjusted for by proximate determinants, with a consequent loss of valuable information related to the causal pathway. Indeed, it is important to explore which underlying determinants can be explained by proximate determinants and which cannot. To do this, the aim should be to report different multivariable analyses with and without underlying determinants to be able to analyze the variations between them. In this review, only 13 studies reported detailed univariable and multivariable analyses, and all of them went from univariable to multivariable, without hierarchy, making generalization of estimates of risk impossible. The STROBE (Strengthening the Reporting of Observational Studies in Epidemiology) statement was published to standardize the reporting of observational studies [25]. It is hoped that by following agreed guidelines, the quality of reporting in observational studies will be more homogeneous, perhaps allowing in the future a generalization of results.

While advocating more consistent reporting, this review highlights the need to move from a rigid etiological analysis to a broader perspective. It is known that gonorrhea infection is sexually transmitted. Therefore, the function of observational epidemiology could be 2-fold: to identify other biological correlates predisposing to infection, such as ectopy, where controlling for confounding is appropriate; and to identify pathways of risk, where excluding variables from analyses because they are correlated would seem inappropriate as the difference in role with and without controlling is informative.

Effectively, the analyses of individual risk factors will always be limited to studying acquisition and duration, rather than spread in the population. But these risks will depend on the population-level risks determining prevalence and thereby exposure. By building a picture of only direct risks, we could overlook variables contributing to the wider spread of infection and potential points for intervention.

## Supplementary Data

Supplementary materials are available at *The Journal of Infectious Diseases* online (http://jid.oxfordjournals.org/). Supplementary materials consist of data provided by the author that are published to benefit the reader. The posted materials are not copyedited. The contents of all supplementary data are the sole responsibility of the authors. Questions or messages regarding errors should be addressed to the author.
